# 真实世界奥布替尼联合R-CHOP方案治疗MCD亚型弥漫大B细胞淋巴瘤疗效及安全性分析

**DOI:** 10.3760/cma.j.cn121090-20240607-00212

**Published:** 2024-09

**Authors:** 华 尹, 玮 华, 浩睿 申, 佳竹 吴, 悦 李, 莉 王, 金花 梁, 建勇 李, 卫 徐

**Affiliations:** 南京医科大学第一附属医院（江苏省人民医院）血液科，南京 210029 The First Affiliated Hospital of Nanjing Medical University (Department of Hematology, Jiangsu Province Hospital), Nanjing 210029, China

**Keywords:** 淋巴瘤，大B细胞，弥漫性, MCD亚型, 奥布替尼, 真实世界, Lymphoma, large B-cell, diffuse, MCD subtype, Orelabrutinib, Real world

## Abstract

**目的:**

探讨奥布替尼联合R-CHOP（OR-CHOP）方案治疗MCD亚型弥漫大B细胞淋巴瘤（DLBCL）患者的疗效及安全性。

**方法:**

对2022年6月至2023年6月共23例在南京医科大学第一附属医院血液科确诊为DLBCL且基线肿瘤组织和（或）基线血浆根据LymphGen算法为MCD亚型的患者进行回顾性分析，患者第1个疗程使用R-CHOP或R-miniCHOP方案，第2～6个疗程使用OR-CHOP或OR-miniCHOP方案（21 d为1个疗程），第7～8个疗程使用利妥昔单抗单药。

**结果:**

23例患者的中位年龄58（30～81）岁，11例（47.8％）年龄>60岁，15例（65.2％）国际预后指数（IPI）评分为3～5分。组织突变频率前10的基因分别为：PIM1（78.3％）、MYD88（69.6％）、ETV6（43.5％）、BTG1（39.1％）、CD79B（43.5％）、HIST1H1E（39.1％）、BTG2（34.8％）、KMT2D（30.4％）、CD58（26.1％）和CDKN2B（21.7％）。组织和血浆基因检测突变的一致率为80％，且基线血浆循环肿瘤DNA（ctDNA）负荷与LDH以及IPI评分密切相关（*P*<0.05）。所有患者均接受5个疗程OR-CHOP方案治疗，中期（3个疗程后）评估总有效率（ORR）为100％（23/23），22例（95.65％）患者获得完全缓解（CR），1例（4.35％）患者获得部分缓解（PR）。治疗结束后ORR为95.65％（22/23），21例（91.30％）患者获得CR，1例（4.35％）患者获得PR，1例（4.35％）患者疾病进展（PD）。21例患者有治疗后血浆ctDNA（EOT-ctDNA）的动态随访结果，4例（19.0％）未达到EOT-ctDNA清零，17例（81.0％）得EOT-ctDNA清零。中位随访时间为20.8（15.3～30.0）个月，中位无进展生存（PFS）和总生存（OS）期均未达到。2年PFS率为71.8％（95％*CI* 54.7％～94.2％），2年OS率为91.3％（95％*CI* 80.5％～100.0％）。此外，OR-CHOP方案临床应用的总体耐受良好，主要不良反应是血液学毒性。

**结论:**

OR-CHOP方案治疗MCD亚型DLBCL患者是安全且存在临床获益的。

## Abstract (en)

**Objective:**

To investigate the efficacy and safety of orelabrutinib combined with R-CHOP in the treatment of MCD subtype diffuse large B cell lymphoma（DLBCL）.

**Methods:**

Twenty-three MCD subtype patients whose gene-subtype classification was based on baseline tumor tissue and/or baseline plasma using the LymphGen algorithm from June 2022 to June 2023 in the First Affiliated Hospital of Nanjing Medical University were retrospectively enrolled in the analysis. All patients were treated with R-CHOP or R-miniCHOP in Course 1, OR-CHOP or OR-miniCHOP（21 days for one course）in Courses 2–6, and R-monotherapy in Courses 7–8.

**Results:**

Of the 23 patients, the median age was 58 years（range: 30–81 years）, and 11（47.8％）aged >60 years. Fifteen cases（65.2％）had international prognostic index（IPI）scores of 3 to 5. The top 10 mutated genes in the gDNA tissues were PIM1（78.3％）, MYD88（69.6％）, ETV6（43.5％）, BTG1（39.1％）, CD79B（43.5％）, HIST1H1E（39.1％）, BTG2（34.8％）, KMT2D（30.4％）, CD58（26.1％）, and CDKN2B（21.7％）. The consistency rate of the tissue and plasma mutations was 80％, while the baseline plasma ctDNA burden was closely correlated with the LDH levels and IPI scores（*P*<0.05）. All patients received 5 courses of OR-CHOP regimens. The mid-term（after 3 courses) evaluation showed that the overall response rate（ORR）was 100%（23/23）, with 22 patients（95.65％）achieving complete remission（CR）, and 1 patient（4.35％）achieving partial remission（PR）. The ORR after the end of treatment（EOT）was 95.65％（22/23）. Moreover, 21 patients（91.30％）obtained CR, 1 patient（4.35％）obtained PR, and 1 patient（4.35％）obtained progression disease（PD）. Of the 21 patients who had the dynamic EOT-ctDNA burden, only four patients（19.0％）did not achieve EOT-ctDNA clearance, while the other 17 patients（81.0％）achieved EOT-ctDNA clearance. The median follow-up time was 20.8（15.3–30.0）months, while the median progression-free survival（PFS）and overall survival（OS）were not reached. The 2-year PFS rate was 71.8％（95％*CI* 54.7％–94.2％）, while the 2-year OS rate was 91.3％（95％*CI* 80.5％–100.0％）. Furthermore, the OR-CHOP regimen was generally well tolerated during clinical use, with hematological toxicity being the main adverse effect.

**Conclusion:**

This study revealed that the OR-CHOP regimen can be used as an effective and safe first-line treatment for MCD subtype DLBCL.

弥漫大B细胞淋巴瘤（DLBCL）是最常见的淋巴瘤亚型，占成人非霍奇金淋巴瘤（NHL）的30％～40％。目前按照LymphGen基因分型可将DLBCL分7种类型，包括MCD亚型、BN2亚型、N1亚型、EZB亚型（进一步分为MYC异常和正常两种亚型）、A53亚型和Other亚型[1]。其中MCD亚型主要特点是具有CD79B突变和MYD88 L265P突变，约占所有DLBCL的8.7％，5年总生存（OS）率仅为40％，预后较差[1]。

在MCD亚型DLBCL中，布鲁顿酪氨酸激酶抑制剂（BTKi）可以通过破坏My-T-BCR超复合物抑制NF-κB信号通路，发挥抗肿瘤作用[2]。Ⅲ期PHOENIX临床试验亚组分析提示[3]，31例年龄低于60岁的MCD亚型DLBCL患者接受伊布替尼联合R-CHOP方案治疗，3年的无事件生存（EFS）率和OS率均为100％。Ⅲ期随机对照GUIDANCE-01研究纳入26例MCD亚型DLBCL，试验组（伊布替尼联合R-CHOP，13例）的总缓解率（CRR）和总有效率（ORR）均优于对照组（R-CHOP组，13例），而治疗相关毒性未见明显增加[4]。以上结果提示在DLBCL患者中，伊布替尼加入一线诱导治疗可显著提高MCD亚型DLBCL的总体有效率和长期生存率。新一代BTKi即奥布替尼具有创新的化合物结构，更小的空间夹角，三维结构与BTK活性中心更匹配，提高了对BTK的抑制活性和选择性。本研究回顾性分析了23例接受奥布替尼联合R-CHOP（OR-CHOP）方案一线诱导治疗的MCD亚型DLBCL患者，评估其疗效及安全性。

## 病例与方法

1. 病例：本研究纳入2022年6月至2023年6月于南京医科大学第一附属医院血液科新诊断为MCD亚型DLBCL的23例患者。纳入标准如下：①根据2016年世界卫生组织造血和淋巴组织肿瘤病理学分类诊断为DLBCL；②肿瘤组织或基线血浆根据LymphGen算法为MCD亚型[1]；③接受OR-CHOP方案进行诱导化疗至少3个疗程，并进行有效评估；④人口统计学数据、基线临床特征和实验室检验资料完整。

2. 资料收集：从医院电子病历系统收集基线人口统计学数据和临床特征，包括性别、年龄、美国东部肿瘤协作组（ECOG）评分、Ann Arbor分期、B症状（发热、盗汗、乏力或体重下降）、LDH、结外累及部位、国际预后指数（IPI）评分［年龄>60岁、Ⅲ～Ⅳ期、ECOG评分≥2分、结外病灶>1个、LDH>正常值上限（ULN）］、影像学资料包括治疗过程中所有18F-FDG PET-CT显像资料。

3. 二代测序（NGS）：基线组织基因组DNA（gDNA）和血浆循环肿瘤DNA（ctDNA）的突变情况以及接受治疗后血浆ctDNA突变的动态变化情况；按照LymphGen基因分型进行基因分型，共检测475个基因，检测方法参照既往发表的文章[5]。

4. 治疗方案：OR-CHOP方案：利妥昔单抗375 mg·m^−2^·d^−1^静脉滴注，第0天；环磷酰胺750 mg·m^−2^·d^−1^静脉滴注，第1天；多柔比星50 mg·m^−2^·d^−1^（多柔比星脂质体30～40 mg·m^−2^·d^−1^）静脉滴注，第1天；长春新碱1.4 mg·m^−2^·d^−1^（最大剂量2 mg/次）静脉推注，第1天；泼尼松60 mg·m^−2^·d^−1^（分2次）口服第1～5天；奥布替尼150 mg/d，第1～21天（第2～6个疗程）；21 d为1个疗程。对于80岁以上老年患者或非常虚弱无法耐受强化疗的患者，使用OR-miniCHOP方案：利妥昔单抗375 mg·m^−2^·d^−1^静脉滴注，第0天；环磷酰胺400 mg·m^−2^·d^−1^静脉滴注，第1天；多柔比星25 mg·m^−2^·d^−1^静脉滴注（多柔比星脂质体15～20 mg·m^−2^·d^−1^），第1天；长春新碱1 mg/次静脉推注，第1天；泼尼松40 mg·m^−2^·d^−1^（分2次）口服，第1～5天；奥布替尼150 mg/d，第1～21天（第2～6个疗程）；21 d为1个疗程。所有患者计划接受1个疗程R-CHOP或R-miniCHOP方案治疗，第2～6个疗程接受OR-CHOP或OR-miniCHOP方案治疗，第7～8个疗程接受利妥昔单抗（R）单药治疗；对中枢神经系统IPI（CNS-IPI）评分4～6分或累及肾脏、肾上腺、睾丸、乳腺、原发腿型DLBCL患者进行中枢神经系统预防［4～8次鞘内注射甲氨蝶呤和（或）阿糖胞苷或2～4个疗程大剂量甲氨蝶呤3.0～3.5 mg·m^−2^·d^−1^］；后续根据患者的年龄制定下一步的计划：年龄>60岁，进入维持治疗；年龄≤60岁，根据年龄调整IPI（aaIPI）决定是否行auto-HSCT。

5. 疗效评估：疗效评估时间点为3个疗程治疗结束后、末次治疗（6个疗程）结束后、治疗结束后的2年内每3个月1次。根据2014版Lugano疗效评定标准，分为完全缓解（CR）、部分缓解（PR）、疾病稳定（SD）和疾病进展（PD）。ORR为CR率和PR率之和。

6. 安全性评价：根据《常见不良反应事件评价标准（CTCAE）》5.0版，对患者每个疗程用药后发生的不良事件（AE）进行分级。

7. 随访：通过住院病历、门诊就诊记录和电话对所有患者进行随访，随访截止日期为2024年6月30日，中位随访时间20.8个月。随访结局事件包括无进展生存（PFS）期和OS期。OS期定义为从确诊至任何原因导致死亡或随访终止的时间。PFS期定义为从确诊至复发或进展的时间。

8. 统计学处理：采用SPSS 26.0和GraphPad Prism 9软件进行统计学分析。患者的临床特征、疗效和AE等资料采用描述性统计分析，生存曲线采用Kaplan-Meier法绘制，统计学相关性采用双侧对数秩和检验。*P*<0.05为差异有统计学意义。

## 结果

1. 临床和分子学特征：共23例初诊DLBCL患者纳入分析，男11例（47.8％），女12例（52.2％）；中位年龄58（30～81）岁，11例（47.8％）年龄>60岁；19例（82.6％）为非生发中心亚型，19例（82.6％）为Ⅲ～Ⅳ期，9例（39.1％）伴有B症状；7例（30.4％）ECOG评分≥2分；15例（65.2％）IPI评分为3～5分；7例（30.4％）患者CNS-IPI评分为4～6分；16例（69.6％）血清LDH>ULN；20例（87.0％）患者累及结外部位，包括骨、骨髓、睾丸、子宫、乳腺、鼻窦、肝脏、胃、心包、胰腺、腮腺等；14例（60.9％）结外病灶>1个，10例（43.5％）骨髓受累。此外，我们发现23例患者组织突变频率前10的基因分别为：PIM1（78.3％）、MYD88（69.6％）、ETV6（43.5％）、BTG1（39.1％）、CD79B（43.5％）、HIST1H1E（39.1％）、BTG2（34.8％）、KMT2D（30.4％）、CD58（26.1％）和CDKN2B（21.7％）；血浆中突变频率前10的基因分别为：PIM1（95.5％）、MYD88（77.3％）、BTG1（50.0％）、CD79B（50.0％）、CDKN2A（50.0％）、HIST1H1E（40.9％）、KMT2D（40.9％）、BTG2（36.4％）、ETV6（31.8％）和CDKN2A（40.9％）。基线患者血浆ctDNA负荷与LDH以及IPI评分密切相关，高LDH水平与较高的基线ctDNA负荷相关，且高IPI评分患者具有较高的基线ctDNA负荷（*P*<0.05）。此外，组织和血浆检测突变的一致率为80％（图1）。

**图1:**
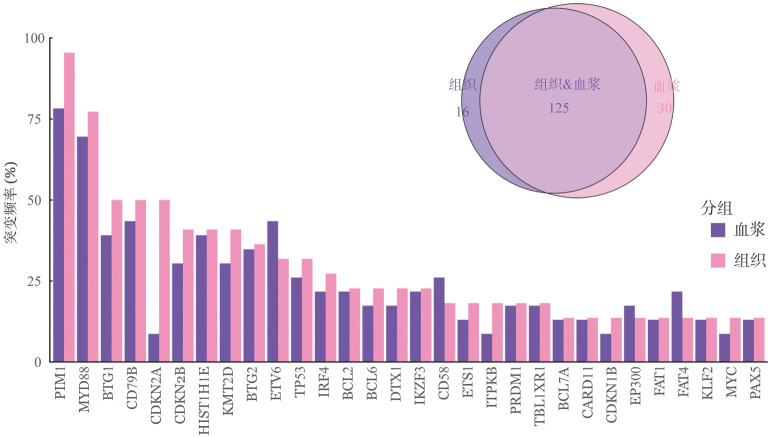
23例接受OR-CHOP样方案治疗的MCD亚型弥漫大B细胞淋巴瘤患者的分子学特征

2. 疗效评价：23例患者均接受1个疗程R-CHOP或R-miniCHOP方案和5个疗程OR-CHOP或OR-miniCHOP方案治疗，22例患者接受了2个疗程R单药治疗；16例患者接受来那度胺维持治疗，3例患者接受了auto-HSCT巩固治疗。按照NCCN指南，12例患者需要进行中枢神经系统预防，其中11例患者完成4～8次鞘内注射，1例患者因既往腰椎手术史行大剂量甲氨蝶呤2个疗程化疗进行中枢神经系统预防。中期（3个疗程后）评估：ORR为100％（23/23），22例（95.65％）患者获得CR，1例（4.35％）患者获得PR。诱导治疗结束后疗效评估：ORR为95.65％（22/23），21例（91.30％）患者获得CR，1例（4.35％）患者获得PR，1例（4.35％）患者PD。中位随访时间为20.8（15.3～30.0）个月，中位PFS和OS期均未达到。2年PFS率为71.8％（95％*CI* 54.7％～94.2％），2年OS率为91.3％（95％*CI* 80.5％～100.0％）。在23例患者中，21例（治疗结束后均为CR）有治疗后血浆ctDNA（EOT-ctDNA）动态随访结果。在这21例患者中，4例（19.0％）患者未达到EOT-ctDNA清零（PET-CT评估均为CR），17例（81.0％）患者取得EOT-ctDNA清零（PET-CT评估16例为CR，1例为PR）。

3. OR-CHOP方案安全性评估：OR-CHOP方案最常见的血液学毒性是中性粒细胞减少（21例，91.3％）。3～4级中性粒细胞计数减少12例（52.2％）、贫血7例（30.4％）、血小板计数降低6例（26.1％）。中性粒细胞减少伴感染6例（20.1％）。最常见的非血液学毒性是肺部感染（8例，34.8％）。其余非血液学毒培养阳性1例（4.3％），粪便真菌培养阳性2例（8.7％），ALT/AST升高3例（13.0％），皮疹2例（8.7％），腹泻1例（4.3％），心律失常1例（4.3％）。3～4级非血液学毒性主要包括肺部感染及心律失常。

## 讨论

本研究为目前所知国内应用奥布替尼联合R-CHOP或R-miniCHOP方案治疗MCD亚型DLBCL最大队列报告。与既往报告的MCD亚型DLBCL临床特征类似[6]–[10]，本研究中绝大多数患者为高危（IPI评分为2～5分，91.3％）且伴有较多的结外病灶的累及（87.0％），提示预后较差。Ⅲ期随机对照GUIDANCE-01研究纳入26例MCD亚型DLBCL患者，试验组（伊布替尼联合R-CHOP）的CR率和ORR均为85％，明显优于对照组（R-CHOP组，CR率为54％，ORR为69％）[4]。Ⅲ期PHOENIX临床试验亚组分析提示，31例年龄低于60岁的接受伊布替尼联合R-CHOP方案的MCD亚型DLBCL患者，3年的EFS率和OS率均为100％[3]。在一项真实世界研究中，18例伴有MYD88/CD79B突变的患者接受泽布替尼或奥布替尼联合R-CHOP方案，ORR为100％，CR率为94.4％[10]。与既往研究类似，在本研究中，23例初诊MCD亚型DLBCL患者经过6个疗程OR-CHOP方案治疗后ORR为95.65％（22/23），21例（91.30％）患者获得CR，1例（4.35％）患者获得PR，1例（4.35％）患者PD。血液学毒性是最常见的不良反应，非血液学毒性主要为肺部感染。提示在一线R-CHOP样方案的基础上联合奥布替尼，可显著提高MCD亚型DLBCL的总体有效率和总体生存。

ctDNA是肿瘤细胞凋亡时释放入外周血的DNA，可真实、完整反映肿瘤组织的整体基因特征；近年来，ctDNA在恶性淋巴瘤尤其是DLBCL中的作用尤为突出，主要用于精准诊断、基因分型、预后预测和疗效监测[11]–[14]。2020年Wright等[1]在2018年提出的“四种基因分型”[15]的基础上进一步提出了可纳入更高比例（63.1％）DLBCL人群的“七种LymphGen基因分型”。然而所有大规模研究的结论均基于全基因组、全外显子测序或大型综合靶向NGS。Zhu等[16]研究发现使用高复杂性检测（如LymphGen）对DLBCL进行的基因组分类，同样可以通过精心设计的较小靶向NGS检测（如IMPACT）应用于常规临床。Meriranta等[11]研究发现，在DLBCL中，基线血浆ctDNA和组织gDNA在基因分型上具有高度的一致性。在本研究中，利用475个基因的panel对gDNA和ctDNA进行基因突变检测，随后进行基线的基因分型和后续的微小残留病的监测。同样发现组织和血浆的基因突变具有较高的一致性，基于组织gDNA的MCD亚型例数为22例（95.6％），而基于血浆ctDNA的MCD亚型例数为18例（78.2％）。在组织分型为MCD亚型而血浆分型未分为MCD亚型的5例患者中，2例患者为血浆ctDNA阴性。此外，既往多项研究已经证实基线ctDNA高负荷与高危的基线临床特征和较差的预后密切相关[12]。在本研究23例MCD亚型DLBCL患者中，我们同样发现较高的基线ctDNA负荷与高LDH和高IPI评分密切相关，提示基线ctDNA负荷与MCD亚型DLBCL患者的预后密切相关。

综上所述，OR-CHOP方案一线治疗MCD亚型DLBCL患者具有良好的耐受性，并且患者的有效率和生存具有较好的结果。由于本研究是回顾性真实世界分析，且为单中心研究，样本量小，需前瞻性多中心研究且扩大样本量来验证OR-CHOP方案的有效性和安全性；此外，也需要随机对照研究（OR-CHOP方案对R-CHOP方案）为奥布替尼一线联合治疗初诊MCD亚型DLBCL提供更强的循证医学证据。
